# Disruption of embryonic ROCK signaling reproduces the sarcomeric phenotype of hypertrophic cardiomyopathy

**DOI:** 10.1172/jci.insight.146654

**Published:** 2020-12-17

**Authors:** Kate E. Bailey, Guy A. MacGowan, Simon Tual-Chalot, Lauren Phillips, Timothy J. Mohun, Deborah J. Henderson, Helen M. Arthur, Simon D. Bamforth, Helen M. Phillips

Original citation: *JCI Insight*. 2019;4(8):e125172. https://doi.org/10.1172/jci.insight.125172

Citation for this retraction: *JCI Insight*. 2020;5(24):e146654. https://doi.org/10.1172/jci.insight.146654

Newcastle University recently notified *JCI Insight* of data manipulation in this article. In accordance with the institutional recommendation and at the request of the corresponding author, *JCI Insight* is retracting this article.

## Version 1 12/17/2020

Electronic publication

